# Supplementary material for: Content-rich biological network constructed by mining PubMed abstracts
Source: BMC Bioinformatics. 2004 Oct 8;5:147. doi: 10.1186/1471-2105-5-147 (PMC528731; doi:10.1186/1471-2105-5-147)
Supplement: Additional File 2 — The original results of the above study (non-essential files are deleted to keep the file size under the limit set by BMC bioinformatics). [file 1471-2105-5-147-S2.bz2 › chilibotAdditionalFile2/dip20/218ID9694899E726/html/IL6R_IL6.html]

 


 **IL6R** and **IL6** 
  
Found 377 abstracts in PubMed, retrieved 20.  
 

 What does Google say? 
 PDF only 
| .edu only 

---

**Interactive relationship** (e.g. stimulation, inhibition, etc)

**Stimulatory relationship**- The mRNAs of both receptors of IL 6  [ **IL6** ] , the soluble  **IL6R**  and gp130, were increased subsequently to IL 6  [ **IL6** ]  mRNA.  Ref: 12733076 Pflugers Arch< MedlineTA>Pflugers Arch,
**Neutral relationship**- The human interleukin 6  [ **IL6** ]  receptor  [ **IL6R** ]   **IL6R**  is responsible for signal transduction of  **IL6**  that might have associations with immune diseases and various infectious diseases.  Ref: 12655571 Hum Mutat, 2003

**Non-interactive relationship** (e.g. studied together, co-existance, homology, etc.)

- An aminopeptidase, ARTS 1, is required for interleukin 6  [ **IL6** ]  receptor  [ **IL6R** ]  shedding.  Ref: 12748171 J Biol Chem< MedlineTA>J Biol Chem, 2003
- Interleukin 6  [ **IL6** ]  receptor  [ **IL6R** ]  expression and localization after transient global ischemia in gerbil hippocampus.  Ref: 12676341 Neurosci Lett, 2003
- Co expression of interleukin 6  [ **IL6** ]  IL 6  [ **IL6** ]  and interleukin 6  [ **IL6** ]  receptor  [ **IL6R** ]  IL 6R in thyroid nodules is associated with co expression of CD30 ligand CD30 receptor.  Ref: 12553555 J Endocrinol Invest, 2002
- Interleukin 6  [ **IL6** ] , soluble interleukin 2 receptor and soluble interleukin 6  [ **IL6** ]  receptor  [ **IL6R** ]  in the sera of patients with different histological patterns of rheumatoid synovitis.  Ref: 12673891 Clin Exp Rheumatol< MedlineTA>Clin Exp Rheumatol, 2002
- Relationship between circulating serum soluble interleukin 6  [ **IL6** ]  receptor  [ **IL6R** ]  and the angiogenic cytokines basic fibroblast growth factor and vascular endothelial growth factor in multiple myeloma.  Ref: 12574959 Ann Hematol, 2003
- Data on the expression of interleukin 6  [ **IL6** ]  IL 6  [ **IL6** ]  interleukin 6  [ **IL6** ]  receptor  [ **IL6R** ]  IL 6R in thyroid nodules is scarce.  Ref: 12553555 J Endocrinol Invest, 2002
- Aim To measure serum concentrations of the soluble interleukin 2 receptor sIL2R, soluble interleukin 4 receptor sIL4R, soluble interleukin 6  [ **IL6** ]  receptor  [ **IL6R** ]  sIL6R, and soluble tumor necrosis factor alpha receptor I and soluble tumor necrosis factor alpha receptor II, during the perinatal and early neonatal period, in order to evaluate their role in activation of immune response in labor and the first days postpartum.  Ref: 12857603 Mediators Inflamm, 2003
- Cellular cholesterol depletion triggers shedding of the human interleukin 6  [ **IL6** ]  receptor  [ **IL6R** ]  by ADAM10 and ADAM17 TACE.  Ref: 12832423 J Biol Chem< MedlineTA>J Biol Chem, 2003
- Increased soluble interleukin 6  [ **IL6** ]  receptor  [ **IL6R** ]  in vitreous fluid of proliferative vitreoretinopathy.  Ref: 12789531 Curr Eye Res, 2003
- Identification of novel SNPs in the interleukin 6  [ **IL6** ]  receptor  [ **IL6R** ]  gene  **IL6R** .  Ref: 12655571 Hum Mutat, 2003
- The interleukin 6  [ **IL6** ]  receptor  [ **IL6R** ]  can serve as an alpha receptor for CTNF.  Ref: 12643274 J Biol Chem, 2003
- Part II Significance of anti interleukin 6  [ **IL6** ]  and anti soluble interleukin 6  [ **IL6** ]  receptor alpha  [ **IL6R** ]  antibodies in a standardized murine contact burn model.  Ref: 12771624 Crit Care Med, 2003
- In vivo activation of the interleukin 6  [ **IL6** ]  receptor  [ **IL6R** ]  gp130 signaling pathway in pituitary corticotropes of lipopolysaccharide treated rats.  Ref: 12624539 Neuroendocrinology, 2003
- Analysis of myeloma cell derived plasma membrane fractions shows that caveolin 1 is co localized with interleukin 6  [ **IL6** ]  receptor  [ **IL6R** ]  signal transducing chain gp130 and with insulin like growth factor I receptor.  Ref: 12482878 J Biol Chem, 2003
- 2 mediators and receptors of immunity, i.e. neopterin, soluble interleukin 2 receptor sIL 2R, soluble interleukin 6  [ **IL6** ]  receptor  [ **IL6R** ]  sIL 6R, soluble tumor necrosis factor receptor sTNF RII.  Ref: 12708230 Int J Hyg Environ Health, 2003
- We have determined the percentage of alphabeta and gammadelta T cells by flow cytometry as well as serum interleukin 6  [ **IL6** ]  IL 6  [ **IL6** ]  and soluble interleukin 6  [ **IL6** ]  receptor  [ **IL6R** ]  sIL 6R levels by enzyme linked immunosorbent assay in kidney allograft recipients with acute, chronic or stable graft evolution.  Ref: 12828564 Scand J Immunol< MedlineTA>Scand J Immunol, 2003
- Replacing the TfR stalk by the corresponding sequences from tumor necrosis factor alpha or interleukin 6  [ **IL6** ]  receptor  [ **IL6R** ]  also led to TfR ectodomain shedding.  Ref: 12586332 FEBS Lett, 2003
- Using the recently engineered designer cytokine IC7, which induces an active heterodimer of the LIFR and gp130 after binding to the IL 6R, and several receptor chimeras of the LIFR and the interleukin 6  [ **IL6** ]  receptor  [ **IL6R** ]  IL 6R carrying the CBM of the IL 6R in place of the COOH terminal LIFR CBM, we could assign individual receptor subdomains to individual binding sites of the ligand.  Ref: 12731865 Biochemistry, 2003
- Polymorphisms of the gene encoding the inflammatory cytokine interleukin 6  [ **IL6** ]  determine the magnitude of the increase in soluble interleukin 6  [ **IL6** ]  receptor  [ **IL6R** ]  levels in Alzheimer s disease Results of a pilot study.  Ref: 12664314 Eur Arch Psychiatry Clin Neurosci, 2003
- Since hydroxamic acid based metalloprotease inhibitors prevent shedding of both TNFR1 and the interleukin 6  [ **IL6** ]  receptor  [ **IL6R** ]  IL 6Ra, we hypothesized that ARTS 1 might also regulate shedding of IL 6Ra, a member of the type I cytokine receptor superfamily that is structurally different from TNFR1.  Ref: 12748171 J Biol Chem< MedlineTA>J Biol Chem, 2003
